# Estimating measurement error in child language assessments administered by daycare educators in large scale intervention studies

**DOI:** 10.1371/journal.pone.0255414

**Published:** 2021-11-19

**Authors:** E. F. Haghish, Werner Vach, Anders Højen, Dorthe Bleses

**Affiliations:** 1 Department of Psychology, PROMENTA Research Center, University of Oslo, Oslo, Norway; 2 Department of Environmental Sciences, University of Basel, Basel, Switzerland; 3 Basel Academy for Quality and Research in Medicine, Basel, Switzerland; 4 TrygFondens Centre for Child Research and School of Communication and Culture, Aarhus University, Aarhus, Denmark; The Education University of Hong Kong, HONG KONG

## Abstract

Measurement error is a ubiquitous element of social science studies. In large-scale effectiveness intervention studies on child language, administration of the assessment of language and preliteracy outcomes by speech and language pathologists is costly in money and human resources. Alternatively, daycare educators can administer the assessment, which preserves considerable resources but may increase the measurement error. Using data from two nationwide child language intervention studies in Denmark, this article evaluates daycare educators’ measurement error when administering a test of language and preliteracy skills of 3 to 5 year old children that in part is used in a national screening program. Since children were randomly assigned to educators, hierarchical linear models can estimate the amount of additional measurement error caused by educators’ language assessments. The result shows that the amount of additional measurement error varied between different language subscales, ranging from 4% to 19%, which can be compensated for by increasing the sample size by the latter percentage. The benefits and risks of having daycare educators administer language assessments are discussed.

## Introduction

One area of development that is highly predictive of children’s future skills as readers and writers is early language and preliteracy development [1]. Researchers have therefore devoted considerable attention to designing and testing interventions for preschoolers to improve their language and literacy skills. However, a large body of evidence for the effects of language and literacy intervention in young children’s language development is based on efficacy trials. As a result, our understanding of whether and to what extent these interventions work when taken to scale is limited [2, 3]. Large-scale intervention studies are known to be extremely costly, demanding substantial amounts of human and monetary resources. Therefore, examining alternative procedures that bring down their costs is key to making them feasible. This is the main idea of the current manuscript, where we demonstrate 1) how language assessments can be carried out by daycare educators in a large-scale study, 2) how well daycare educators can perform language assessments, and 3) how can we compensate for the measurement errors added by the daycare educators.

One of the major costs of studies of early language and preliteracy intervention is the assessment of language outcomes. Typically a battery of standardized test is administered by either speech and language pathologist or by trained research assistants [4–6]. Consequently, as the sample size increases, having speech pathologists or research assistants carry out the assessment of outcomes becomes costly. The purpose of the current study is to assess measurement error in daycare providers’ administration of a standardized language and preliteracy tool prior to an intervention study and, in turn, shed light on the validity of substituting daycare providers for trained speech and language pathologists or research assistants in large-scale intervention trials.

To put the current study into perspective, it is vital to note that in recent years, there has been emerging policies in several countries to assess children’s language and preliteracy skills in preschool and daycare centers. This is done partly because of increasing number of bilingual children who struggle with acquiring the majority language alongside the content of the curriculum [7] and the serious concequences that lack of language proficiency can have on children’s education. The purpose of these assessments is to identify children at risk for delayed language development and to accelerate language acquisition in classrooms by allowing educators to target instructional activities to individual children’s needs [8, 9]. Recent policies in Denmark, where the current research was carried out, require the daycare centers to administer a program for assessing language and preliteracy skills of three-year-old children. The purpose of the program is to identify delayed children and initiate language support for them, while also paying attention to the needs of well-developed children.

Daycare educators’ familiarity with administering this language assessment provides the possibility of involving daycare educators in large-scale intervention studies to measure children’s outcomes, reducing assessment costs compared to outcome measures provided by speech and language pathologists. In Denmark, SPELL (“Structured Preschool Effort for Language and Literacy”) and LEAP (“Language Education Activities for Preschoolers”) are two independent large-scale randomized control trials of language and literacy interventions, each involving about 7,000 children in more than 300 daycare centers. The children’s daycare educators performed the pre- and posttests language and preliteracy assessment serving as pre- and posttests with a battery of structured language assessment tools in Danish language [for more information regarding the projects see 10–12].

Given the large size of these intervention studies, using speech and language pathologist or research assistants for administering the outcome measures was not economically feasible. Instead, the daycare educators were asked to conduct the pre- and posttests. Despite the practical efficiency of using daycare educators for carrying out the language assessments, little is known about the reliability of their assessments and the measurement error attributable to educators [13]. In other words, when educators are asked to administer the assessment, children’s scores will not only reflect their individual differences, but also individual differences between educators, which is commonly referred to as *assessors variance*, *assessor bias*, or *assessor measurement error*. Accounting for this variance is crucial because it is a threat to the validity of the assessments [14]. Moreover, having an estimation of the amount of assessors’ variance, especially when school teachers or daycare educators are asked to carry out the assessments, can facilitate their further engagement in large-scale studies.

Recently, there has been a few studies (we only found two) addressing assessors’ variance using Hierarchical Linear Models (HLM) in large studies. For example, McDermott et al., (2014) evaluated the school psychologists’ bias variance when administering Wechsler Inteligence Scale for Children on children rangeing in age between 6–16 (M = 10.3 years, SD = 2.5). After controlling for demographic variables (conditional model), depending on the subscale, the estimated assessors’ variance ranged from 2.4% (*p*>0.05) to 12.5% (p < 0.001). Interestingly, the highest amount of assessors’ variance was reported for the three subscales of Wechsler’s Verbal Comprehension Index, which are similarities (10%, p < 0.001), vocabulary (7.4%, p < 0.001), and comprehension (9.9%, p < 0.001). However, estimating school psychologists measurement error for IQ tests is probably more comparable to speech therapists rather than school teachers, when it comes to languages assessments. In a similar manner, Waterman et al. [13, 15], explored assessors’ variance among preschool teachers and extramural assessors for various tests and concluded that preschool teachers had dramatically higher variances while the variances of the extramural assessors were neglegible.

We argue that there are problems with both studies that can bias their results. For example, both of these studies used a relatively simple model with two hierarchical levels by specifying the teachers/assessors as the only random effect parameter (Waterman et al., 2012 also reports an analysis with 3 levels), which may not yield an accurate estimation of the assessors’ variance. There might be large variances resulting at other levels, such as classroom, institute, or even municipalities that will not be accounted by a two-level model and bias the assessors’ variance estimation. Furthermore, none of the studies reported any randomized procedure for assignment of children to an assessor, which complicates the interpretation of the analysis. In the next section, we provide a detailed explanation to address this point.

Similar to the studies mentioned above, in the current article we apply hierarchical linear models to determine the amount of measurement error attributed to daycare educators for assessing children’s language skills in a large-scale intervention study. However, we make addition use of a random assignment of groups of children to educators and will discuss why doing so is important for evaluating the assessors’ variance. In doing so, we will model the hierarchical structure of the data as accurate as possible to reflect on the complete hierarchical structure of the data. By doing so, we explicitly determine variance inflation factors describing the additional variance we have to compensate for in designing a study. In contrast, we will also explore the effect of model misspecification for evaluating assessors’ variance—as carried out by the studies mentioned above–and compare the results with the accurate model. We will discuss under what circumstances this method can be used and what aspects should be taken into account when interpreting the assessor variance.

### Measurement error estimation in HLM

**Fig 1:**
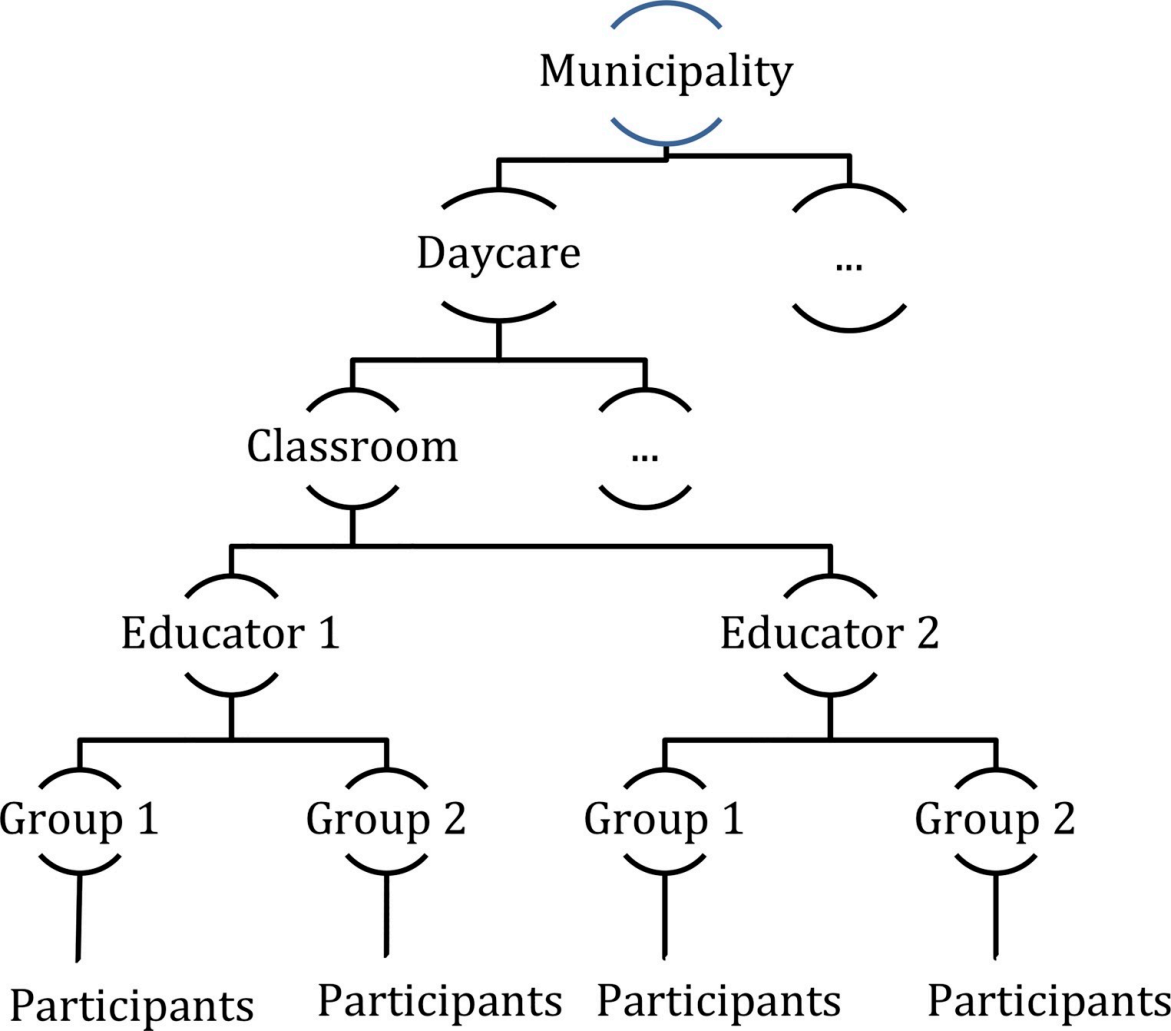
The hierarchical structure of the analyzed data sets.

The hierarchical structure is a characteristic of organizations where individuals form groups within the organization. The data collected from hierarchical organizations will also have a hierarchical structure. The data of the current study came from two large-scale effectiveness studies carried out in thirteen Danish municipalities. Within each municipality, several daycare centers were included and all children of the centers between 3 and 5 years old were included in the study. Each daycare had several classrooms each with typically two educators, and finally, each educator had to perform language assessments in two groups of children, which were randomly assigned to each educator. In this example, the rather deep nesting structure is children within groups within educators within classroom within daycares within municipalities. Fig 1 illustrates the nesting structure of the SPELL and LEAP projects.

A principal problem of hierarchical data structure that makes traditional statistical methods unsuitable is that nested observations (e.g. children nested in daycares with different socioeconomic profiles) tend to correlate and consequently violate the assumption of independent observations [16]. In our example, language assessments might also be dependent on the daycare educators. One educator might be very conservative in his assessment while a less-conservative assessor might evaluate the same child slightly higher. Ignoring the hierarchical structure would result in aggregation bias, incorrect standard errors, and consequently imprecise p-values [17].

Hierarchical Linear Models (HLM), also known as mixed models, are common tools in the social sciences to address a hierarchical data structure [18]. These models have two main qualities that make them suitable for the current analysis. Firstly, they take into consideration that individuals within groups tend to be more comparable than between groups. In addition, they can inspect variance of the outcome measure at several unit levels [19]. Besides, it is well known that hierarchical models can be used in order to estimate the measurement error when some measures are replicated [20]. In our context, we do not have true replicates, but we can make use of the fact that children were randomly assigned to educators. The logic of such an analysis is to begin with a simple model explaining the measurement error. For this example, only two levels are included, namely children (*i*) and the daycare educators (*j*), who carried out the language assessments. Therefore, each educator (*j*_1_,*j*_2_,*j*_3_,…) will have several children (*i*) associated with the educator level (*i*_1_*j*_1_, *i*_2_*j*_1_,…,*i*_*n*_*j*_1_). Then, *Y*_*ij*_ denoting the observed language score of the child *i* obtained by the educator *j* is equal to *X*_*ij*_+*ξ*_*ij*_, which is the true value of the child *X*_*ij*_ plus the measurement error *ξ*_*ij*_, respectively [21]:

$$Y_{ij}=X_{ij}+\xi_{ij}$$

The measurement error *ξ*_*ij*_ can be broken into two separate components, *ζ*_*j*_ which presents the typical level of the measurement error of educator *j* and *ϵ*_*ij*_ which indicates the child-specific measurement error and is unique for each child. By definition, we have E(*ϵ*_*ij*_) = 0 and also that *ζ*_*j*_ and *ϵ*_*ij*_ are not correlated. Our interest is the variance of *ζ*_*j*_, i.e. the variance between educators reflecting that some educators may tend to score higher and some tend to score lower. Combining these notations with the previous equation, the observed value *Y*_*ij*_ can be expressed as:

$$Y_{ij}=X_{ij}+\zeta_{j}+\epsilon_{ij}$$

However, the true value of *X*_*ij*_ is unknown and cannot be distinguished from *ϵ*_*ij*_. Therefore, the hierarchical linear model to be fitted is actually:

$$Y_{ij}=\zeta_{j}+{\overset{´}{\epsilon }}_{ij}$$

With

$${\overset{´}{\epsilon }}_{ij}=X_{ij}+\epsilon_{ij}$$

It can only be expected that fitting this model results in a correct estimate of the variance of *ζ*_*j*_ if *ζ*_*j*_ and ${\overset{´}{\epsilon }}_{ij}$ are uncorrelated. Without a randomized assignment of children to the educators, this assumption cannot be made. For example, educators who tend to give high scores might also select poor performing children or prefer selecting high performing children implying a negative or positive correlation, respectively. Randomization insures that *X*_*ij*_ and *ζ*_*j*_ are uncorrelated and, consequently, that also ${\overset{´}{\epsilon }}_{ij}$ and *ζ*_*j*_ are uncorrelated.

Therefore, by fitting an HLM we obtain two estimated measurement error variances: subject-specific measurement error, which appears as residual variance, and level-2 measurement error, which is introduced by the daycare educators. Extending the idea to the full model shown in Fig 1, we can then define the Variance Inflation Factor (VIF) by calculating the fraction of daycare educator variance to other random effect variances obtained from the HLM.


$$VIF=\frac{Var(Daycare\;Educator)}{Var(Municipality)+Var(Daycare\;Center)+Var(Room)+Var(Group)+Var(Residual)}$$

The variance inflation factor reflects how much the variance of a single measurement (after adjustment for fixed effects) which is equal to the sum of variances of all levels (i.e. daycare center, room, educator, group, and residual) is increased compared to the variance in an ideal situation where the educators’ variance equals zero. Similar concepts of VIFs can be found as measures of collinearity [22], where the VIF indicates to what extent the variance has been inflated due to lack of independence between the independent variables in the model [23].

However, to make our results comparable to previous studies [e.g. 13], we also report the relative variance, which is simply the percentage of the daycare educators’ variance compared to the overall variance. As a result, the relative variance is smaller than the VIF.

## Method

### Background

In Danish daycare centers, daycare educators are to some extent familiar with the language assessment tools. However, they do not receive any formal training on language testing. When daycare educators are asked to carry out language assessments in a large-scale effectiveness study, the first question that comes to mind is whether they can efficiently perform language assessments. Having an idea about the measurement error of daycare educators relative to amount of measurement error of speech and language pathologists would provide crucial information for optimizing a large-scale study. However, our knowledge on the amount of the educatrs’ measurement error as well as speech and language pathologists is very limited [13]. Furthermore, answering this question–in a common test-retest fashion–surely requires a study of its own and there is indeed much research needed to through some light on this matter.

### Study aim

In the current manuscript, we follow two main ideas. First, we will approach the same research question mentioned above i.e. how well daycare educators can perform language assessment in Danish daycare centers. We will examine the measurement error of the daycare educators for different language subscales using real-world data from two large-scale studies. In doing so, we will model the random effect parameters as accurate as possibly, by including 6 higherarchical levels (municipality, daycare center, classroom, educator, randomly assigned groups, and children) in the model. We will also repeat the analysis for misspecified models with only two levels (educators and children) and expect higher VIF values for educators. The second aim of the study is to examine the correlation between the daycare educators’ measurement errors across different language subscales. Finally, we will discuss our findings along with other pros and cons of using daycare educators for carrying out language assessments in large-scale studies.

### Data and participants

The data of the current study came from the previously mentioned SPELL and LEAP projects. Both are cluster-randomized trial studies, aiming to evaluate the effectiveness of language and literacy interventions for three to five-year old children in a real-world setting. In both of these studies, children’s language proficiency was assessed by the educators prior to the interventions. Therefore, we used the pre-test scores of both studies in the current study. More specifically, our analysis is based on a subset of the baseline (pre-test) measurements of all children who participated in a group-based instruction and were randomly assigned to an educator who performed the baseline assessment as well as the intervention. 5,759 children (48% female and 11% bilingual), range in age from 36 to 71 months (mean = 53.3 and SD = 9.95) from 14 municipalities were included in the analyses of the current article. The data of SPELL and LEAP projects included two measurements, a pre-test, administered prior to the intervention and a post-test, administered after the intervention. As noted, the analysis reported is solely based on the pre-test.

As explained at the outset of the article, in addition to the municipality, the data included four additional hierarchical levels, i.e. 168 daycare centers, 412 classrooms, 694 educators, and 1245 groups. Each daycare center included several classrooms each with typically two educators. The children of each classroom were grouped by the head of the daycare center into subgroups of about five children. It was allowed to aim at group homogeneity with respect to language performance and age. Finally, groups of children were randomly assigned to each daycare educator. In practice, the number of educators per classroom and the number of groups assigned to each educator varied slightly; 143 educators (20%) were randomly assigned to just a single group since some classrooms in the daycare centers were too small.

Overall, the nested structure of the data included six levels, which also demands a larger dataset compared to single-level models [24]. More importantly, HLM models are known to require reasonable sample size at each level of the hierarchical model [25]. The original sample size calculation for evaluating the interventions’ effect noted the need for 128 daycare centers with an average of three classrooms per daycare and 20 children/2 educators per classroomresulting in 384 classrooms [12]. Our analysis, however, included even larger dataset at daycare and classroom levels. In addition, based on rough stimations of sample sizes of large-scale assessments (LSA), which often include more than 4000 students with at least 20–30 individuals per cluster, the data used in the current analysis meets the criteria [26].

### Ethics

As noted above, the data of the current study is coming from SPELL and LEAP projects, but only include the language assessments that were carried out prior to the intervention. Due to the registration with the Danish Data Protection Agency, the SPELL and LEAP projects were categorised as public research and were approved to be carried out nation-wide in Denmark. For public research projects of significant societal importance–such as SPELL and LEAP—it is not required to ask for consent from each parent in Denmark, because the language assessment is a part of the daycare centers’ activities. For more information, see the published study protocol that explains the ethical approval and all of the privacy procedures implemented in the study [12].

### Open science

The analysis code is documented and is publically available via https://osf.io/gp7z9/. Although the data set is not available publically due to the privacy concerns, the analysis is prepared in reproducible manners and all decisions that were carried out on the data are documented. Reproducible, in this context, implies that the entire analysis can be re-executed (assuming availability of the data) without any help or clarifications from the authors [27].

### Language assessments

Children’s initial language skills was assessed using a revised version of the *Language assessment of children*: 3–6 tool, which includes two sets of age-dependent language and preliteracy subscales for 3 and 4-6-year-old children [28]. The tool is routinely used by daycare educators for language screening of children in daycare centers of most municipalities in Denmark as a part of a national language screening program. Only one of the participating municipalities routinely used a different tool, and participating daycare educators from that municipality received training in a one-day workshop on using the current assessment tool.

For 3-year-old children, the tool consists of a battery of four language and preliteracy subscales, *viz*. Sound Discrimination, Productive Vocabulary, Comprehension, and Communication (see Table 2). The 4-6-year-old language test consists of a battery of six subscales, *viz*. Rhyme Detection, Deletion, Letter Identification, Comprehension, Communication, and Productive Vocabulary. Table 1 shows the number of items, score range, as well as mean score and number of tested children for each language scale and Table 2 describes the subscales briefly.

**Table 1:** Number of items, score range, mean score and number of participants for each language scale.

Language Subscales	N. of Items	Range	Mean Score by Age			N. of Participants
			3 year	4 year	5 year	
**Communication**	10	10–40	30.18	27.06	29.27	5667
**Productive Vocabulary**	76	0–76	26.33	44.26	55.65	5707
**Sound Discrimination**	16	0–16	14.77	-	-	1805
**Comprehension (3-y)**	20	0–20	15.25	-	-	1811
**Comprehension (4-6-y)**	27	0–27	-	17.48	19.96	3857
**Letter Identification**	12	0–12	-	5.63	7.79	3614
**Rhyme Detection**	17	0–17	-	9.31	11.29	3299
**Deletion** *	20	0–20	-	1.99	5.34	3781

* The Deletion subscale included two training items. If the child failed on these training items, the subscale was not administered and the child received a score of 0.

**Table 2:** Description of the tasks involved in subscales of the language assessment instrument.

Language Subscales	Age	Description
**Communication**	3	A communicative situation is described and the educator rates how often the situation occurs (i.e. never, seldom, often, or always). For example, the educator is asked how often the child asks about the meaning of a particular word while the educator reads a particular story book.
**Sound discrimination**	3	The child is shown 2 pictures, each including an object. The name of the objects differed in the initial consonant (e.g. “*hus”* and “*mus"* which are house and mouse in Danish respectively). The educator says the names of both objects, followed by repeating one of them loudly. The child is asked to point to the related picture.
**Comprehension**	3	The child is shown several toys (e.g. a boy, girl, elephant, fish, etc.). Then the child is asked to manipulate the toys based on instructions given by the educator.
**Vocabulary**	3 to 6	The child is asked to name what is shown to the child in a picture. Some of the items required the educator to show a picture first with a brief statement of what is seen in the picture. The educator, then shows a second picture and ask what is different in the second picture. For example, in one of the items a picture of a man driving a car is shown to the child followed by a statement “this man is driving” and on the next picture, a picture of a man in a boat was shown and the child was asked “what is this man doing. In both types of tasks, a list of acceptable responses (words and phrases) as alternatives to the exact target was provided for each item.
**Comprehension**	4 to 6	The child is shown 4 pictures. Then the educator asks the child to point to a particular object or action in one of the pictures by naming the object or actions/respectively. For example, one of the items asks the child to point to the picture where a man is talking to a woman who is carrying a boy.
**Letter Identification**	4 to 6	The child is shown 5 letters. The educator names one letter and the child is asked to point to that letter.
**Rhyme Detection**	4 to 6	The child is told 3 words while referencing to a picture for each word. Only 2 of the words rhyme. The child should point out the picture corresponding to the word that does not rhyme with the other 2 words.
**Deletion**	4 to 6	The child is asked to say a specific word without parts of the word (word, syllable, sound). For example, the child is asked to pronounce “*banana”* without the first syllable “*ba”*.

## Results

To estimate the measurement error of daycare educators’ language assessments, a Hierarchical Linear Model (HLM) was fitted for each language subscale using the mixed command in Stata 13.1. The models included the age and gender of the children as fixed effects (i.e. independent predictors) and municipalities, daycare center, classroom, educator, and groups as random effects. For the Comprehension and Communication subscales, the analysis was carried out by adding another binary fixed effect predictor that specifies the different tests for 3- and 4–5 year-olds, respectively.

The HLM models were run for each language subscale individually and the estimated standard deviation for the educator along with the confidence interval was extracted from the random-effect tables. In the next step, we computed the relative variance and the variance inflation factor. Table 3 presents information about the educators variance. As expected, the results reveal that both age is a significant predictor of children’s performance for all subscales (p < 0.001). In addition, apart from the deletion subscale, for all other language subscales gender effect favoring girls was also a significant predictor (p < 0.001).

**Table 3:** Estimated SD, relative variance, and VIF assigned to educators.

Language Subscales	Estimated SD	95% CI	Relative Var.	VIF
**Sound Discrimination**	0.84	0.68–1.03	15.7%	18.7%
**Communication**	1.45	1.07–1.96	5.9%	6.2%
**Vocabulary**	4.04	3.32–4.91	7.3%	7.9%
**Letter Identification**	0.67	0.40–1.14	3.9%	4.1%
**Rhyme Detection**	1.55	1.28–1.88	16.3%	19.4%
**Comprehension**	1.47	1.28–1.69	14.6%	17.1%
**Deletion**	1.26	0.99–1.60	6.8%	7.3%

*Note*. The estimated SDs are not directly comparable since the subscales vary in number of items and consequently, the score range and the mean.

We can also observe variance inflation factors for educators ranging between 4.1% and 19.4%, indicating that daycare educators measurement error considerably varies based on the subscales. The subscales seemed to group in two according to the VIF. The high VIF values (17.1–19.4%) were observed for Sound Discrimination, Rhyme Detection, and Comprehension. The small VIF values (4.1–7.9%) were observed for Communication, Vocabulary, Deletion, and Letter Identification.

The low VIF for the Letter Identification may reflect that pointing to a letter does not open many opportunities for an individual interpretation of the test results, which results in less measurement error. The low VIF for Deletion subscale is probably due to the floor effect in this scale, as 56.5% of the children score 0 due to failing the first two test items, and it may be easy for different educators to agree that a child cannot perform this task at all. Omitting the children who failed on the two test items increased the VIF to 10%. The low VIF for the Vocabulary score was somewhat surprising for us, as in this task the educators may take the freedom to accept responses similar to those included in the list of acceptable responses. Hence, this result suggests that providing a list of acceptable responses is helpful to diminish an undesired influence by the teacher.

For Sound Discrimination (administered to 3-year-old children), Comprehension, and Rhyme Detection subscales, we have relatively large VIFs, although educators just had to decide whether the child is pointing to the correct picture, similar to the Letter Identification subscale. For Comprehension and Rhyme Detection, perhaps, this is related to the fact that these tasks are actually more complex to perform for both the educator and the child. For Sound Discrimination, however, there is a substantial ceiling effect. The score of this scale ranges from 0 to 16, however, 10% of the children scored 14, 15.5% scored 15, and 57.5% scored 16, which adds up to 83% of the participants scoring very high on this scale. Thus, the estimated educator VIF for Sound Descrimination should be interpreted with caution.

To further examine the effect of hierarchical model misspecification for random effect parameters, we repeated the analysis with the same independent predictors. However, we defined only two levels of children and educators for the hierarchical model. As shown in Table 4, the model misspecification dramatically incleases the VIF estimates for the educators.

**Table 4:** Estimated SD, relative variance, and VIF assigned to educators for a misspecified model with only two levels.

Language Subscales	Estimated SD	95% CI	Relative Var.	VIF
**Sound Discrimination**	0.95	0.82–1.10	21.1%	26.7%
**Communication**	2.94	2.73–3.17	25.5%	34.2%
**Vocabulary**	6.88	6.39–7.42	26.0%	35.1%
**Letter Identification**	1.22	1.08–1.439	13.0%	14.9%
**Rhyme Detection**	2.03	1.86–2.22	28.8%	40.4%
**Comprehension**	1.95	1.82–2.10	28.9%	40.6%
**Deletion**	1.78	1.59–2.00	13.6%	15.7%

*Note*. The estimated SDs are not directly comparable since the subscales vary in number of items and consequently, the score range and the mean.

To address the question whether educators’ measurement error correlates between the subscales, joint HLMs for each pair of the subscales were fitted allowing to estimate correlations between random effects. The analysis failed for calculating correlations between Sound Discrimination subscale and other language subscales due to the small number of participants for this subscale. Table 5 reports the correlation coefficients with 95% confidence intervals. Not surprisingly, we found the largest correlation between Comprehension and Rhyme Detection subscales, which have the largest VIF according to Table 3. Vocabulary and Comprehension were positively and significantly correlated to each other and to the other two test-based subscales (in contrast to Communication subscale, which is based on educators’ evaluation), indicating a common tendency across these subscales to score too positive or too negative in test-based tasks. The educators did not seem to apply this tendency when answering the Communication subscale, which is based on the educators’ experience with the child. Overall, the positive correlations between all but the Communication subscale indicate that the daycare educators, whether they were too strict with scoring of the subscales’ items (i.e. rejecting borderline responses) or alternatively, too permissive in their evaluations, were inclined to score with similar tendencies across all test-based subscales.

**Table 5:** Correlation coefficients between educators’ random effects.

Subscales	Comprehension	Rhyme	Letter Identification	Vocabulary	Deletion
**Communication**	.00 [-.26.26]	-.05 [-.26.36]	-.36 [-.78.28]	-.16 [-.50.21]	-.28 [-.64.17]
**Deletion**	.20 [-.06.44]	.28 [.00.53]	.15 [-.36.59]	.24 [-.10.53]	
**Vocabulary**	.34 [.16.56]	.36 [.09.58]	.53 [.02.82]		
**Letter Identification**	.30 [-.04.58]	.18 [-.25.56]			
**Rhyme Detection**	.64 [.48.76]				

*Note*. The numbers in brackets present the 95% confidence intervals.

## Discussion

Large intervention studies require a considerable amount of resources, and finding strategies to reduce the costs of implementation can facilitate conducting such studies. In this article, we investigated daycare educators’ measurement error when administering language assessments of 3-5-year-old children as pre- and post-tests. Alternatively, such a task could be done by speech and language pathologists or trained research assistants, which would typically be more expensive. However, having daycare educators administer the assessment of the outcome measures might result in high levels of measurement error compared to experienced speech and language pathologist s, as shown by previous research [13].

We used data from two large-scale cluster-randomized trial studies that intended to evaluate effectiveness of a language and literacy intervention for 3 to 5-year-old in a real-world daycare setting. Our analysis shows relatively low levels of measurement error in educators. The variance inflation factor ranged between 4.1% and 19.4% for different language subscales. To some extent, it seems that the variation in the measurement error can be explained by the degree of subjectivity of the scoring procedure. For example, scoring Letter Identification task is rather objective (i.e. deciding whether the child points to the correct letter) and does not require much interpretation, and consequently we observed a low additional measurement error of about 4.1%. Gamaroff [29] points out that the assessors’ reliability in language assessment is of particular important in subjective tests because the fluctuations in judgement appear between different raters and within the same rater, casting doubt about the *interrater* and *intrarater* reliabilities, respectively. Our analysis also revealed a correlation between the measurement error across the test-based language subscales suggesting that daycare educators who are either relatively strict or more permissive in scoring a particular language subscale, have a similar tendency while scoring other subscales.

In certain respects, our analysis differs from those of previous attempts, namely Waterman et al., [13] and McDermott et al., [15]. On the one hand, participants were randomly assigned to each educator, and on the other, our analysis modeled other influential hierarchical levels that could contribute to the measurement error such as classroom, daycare center, and municipality. Therefore, we believe, our analysis provides a better estimation of the educators’ measurement error. For example, if we repeat our analysis with only 2 levels (i.e. participants and educators) and neglect the other random factors, the measurement error of the educators is incorrectly estimated to be twice the values reported in Table 3. The measurement error–referred to in other studies as “assessor variance” [13] or “assessor bias” [15] respectively–might not merely reflect the measurement errors of assessors, but a combination of measurement error and other random-effect parameters, which consequently over estimate it. Probably, this can also explain why all of the assessor variance reported for different subscales in Waterman et. al. [13] are considerably higher than what we have reported in the current article. Interestingly in McDermott et al., [15] the relative variance of IQ assessments administered by school psychologists is comparable to our results.

Our approach for evaluating measurement error is restricted in several ways. First and foremost, it can only be applied to studies in which participants–or, in our example, groups of participants–are randomly assigned to the assessor. Otherwise, it would not be possible to make an inference about the measurement error. Furthermore, our approach can only estimate the amount of measurement error after baseline data collection and not prior to it. Therefore, the results cannot be used in designing a single study. However, the value of our results lies in providing a general estimate of measurement error in daycare educators’ administration of a standardized test battery. Unfortunately, we do not know of a study that permitted the estimation of measurement error in specialist assessors administrating equivalent standardized tests. However, at least for four of the seven subscales with VIFs of 7.9% or less, the use of specialist assessors could have only marginally reduced measurement error compared to that of the daycare educators of the present study.

We conclude that using daycare educators for pre- and posttesting of children in intervention studies seems valid, even if using specialist assessors might result in marginally smaller measurement error. However, we remind the reader that more cross-cultural research is needed before this conclusion can be extended to other countries. Our results, however, are compatible with Mashburn, Downer, Hamre, Justice, & Pianta [30], reporting that teachers—whom had experience with standardized testing procedures–showed adequate validity and reliability in administering the assessments (note that the assessment tools were different). Once an educated guess can be made, the expected amount of additional measurement error can be rectified by increasing the sample size [31]. For example, we can compensate for an increase in variance by increasing the number of units at the lowest level–i.e. municipality or daycare center–exactly as indicated by the VIF.

If daycare educators carry out the testing poorly and increasing the sample size requires a significant amount of additional resources, this approach will not be practical. However, when there is no reason to believe that using daycare educators (or school teachers) as assessors introduces a large amount of measurement error–as indicated by our results–increasing the sample size can be a reasonable strategy, if the costs of increasing the sample size are relatively low. Particularly, if there is an estimate of how well daycare educators perform language assessments to calculate the necessary increase of the sample size. To be precise, the cost of using speech and language pathologist s or research assistants for carrying out the assessments should be compared and balanced with the cost of increasing the study sample size when the examiners are not well-trained and a higher variance is expected between examiners [32]. As we can also expect some variation among speech and language pathologists, the VIFs presented may overestimate the necessary increase in sample size. In addition, it is also reasonable to assume that daycare educators’ performance can be further improved by providing instructional materials or training courses.

One limitation of our analysis is the use of pretest measurements. In contrast, the analysis of intervention studies is often based on change scores between pretest and posttest. Consequently, sample size considerations have to be based on the variance of change scores. However, if we apply our methodology to change scores, differences between educators may not only reflect the additional measurement error, but also differences in how well different educators in different daycare centers implemented the intervention. Nevertheless, we repeated the analysis for the change scores and we obtained VIFs with similar magnitude, ranging between 10% for Vocabulary and Rhyme subscales to 17% for the Deletion subscale, with exception of Sounds Discrimination subscale which yielded a VIF of 28%.

Furthermore, in the SPELL and LEAP intervention studies, not all children were tested by the educator performing the intervention. Some children were tested by a person in the daycare with special language-related responsibilities or by another daycare educator due to unknown (but likely organizational) reasons. However, only the subset of the original data was used in the current analysis where children were randomly assigned to educators performing the intervention. Therefore, the sample of children included may not be fully representative.

Finally, there might be differences between daycare educators and speech and language pathologists in administering language assessments which are not measured by an increase of the measurement error. For example, educators might be more influenced by remembering pretest values compared to speech and language pathologists, just as they have to test a smaller number of children. More research is required to clarify whether there is such a tendency among daycare educators in administering the assessments and if yes, how large is the magnitude.

To date, we do not know how much measurement error can we expect from speech therapists if hundreds of them administer language assessments on preschool children in a nation-wide study. Such a results would clarify the missing half of the overall picture i.e. how well daycare educators perform the assessments. We also assumed that the higher VIF of the Sound Discrimination, Rhyme Detection, and Comprehension is related to the complexity of performing these tasks in Danish language (for both educators and children). However, there is a need for more research to examin this assumption.
