# Correction: Estimating measurement error in child language assessments administered by daycare educators in large scale intervention studies

**DOI:** 10.1371/journal.pone.0304884

**Published:** 2024-05-31

**Authors:** The PLOS ONE Staff

In the Author Contributions, Anders Højen and Dorthe Bleses should also be listed as people who wrote (review & editing) the paper. The correct contributions are:

**Writing–review & editing:** E. F. Haghish, Werner Vach, Anders Højen, Dorthe Bleses.

The publisher apologizes for the error.
